# The impact of integrative neuromuscular training on the physical fitness of elite male martial arts Sanda Athletes

**DOI:** 10.1371/journal.pone.0349164

**Published:** 2026-05-18

**Authors:** Zhenxing Li, Jia Sun, Rozaireen Muszali, Liang Li

**Affiliations:** 1 Guangdong Justice Police Vocational College, Guangzhou, China; 2 Faculty of Pedagogy, Sherkhan Murtaza University, Taraz, Kazakhstan; 3 Linyi Sanhekou Xiao Xue, Linyi, China; 4 Faculty of Sport Science and Coaching, Sultan Idris Education University, Tanjung Malin, Malaysia; 5 Public Physical Education Department, Linyi City Vocational College, Linyi, China; 6 China Institute of Sport and Health Science, Beijing Sport University, Beijing, China; University of Belgrade: Univerzitet u Beogradu, SERBIA

## Abstract

**Objective:**

Integrative Neuromuscular Training (INT) is recognized not only for its potential in preventing sports injuries but also for enhancing athletic performance. Understanding the relationship between INT and the physical qualities of elite Sanda athletes is important for addressing performance limitations in elite Sanda athletes. While INT has been applied in various sports, there is a lack of research in the context of Sanda. This empirical study aims to investigate the effects of INT on the physical qualities of elite Sanda athletes.

**Methods:**

A randomized controlled trial design was employed, with 26 elite male Sanda athletes randomly assigned to either the Integrative Neuromuscular Training group (INT, n = 13) or the control group (CON, n = 13). The INT group engaged in three sessions of INT training per week, lasting 45 minutes each for 12 weeks, while the CON group completed a conventional conditioning program. Pre- and post-intervention assessments included one-repetition maximum tests (1RM), 30-meter sprint tests (30MST), counter movement jump tests (CMJ), reaction agility tests (RAT), and Y balance tests (YBT).

**Results:**

Post-intervention, the INT group showed significant improvements in these physical fitness measures: [1RM bench press (p < 0.001, d = 2.599), 1RM squat (p < 0.001, d = 2.610), T-5m (p = 0.011, d = −1.000), T-10m (p = 0.011, d = −0.833), T-30m (p < 0.001, d = −6.120), CMJ (p < 0.001, d = 4.236), RAT (p < 0.001, d = −3.312), YBT-L (p < 0.001, d = 3.075), YBT-R (p < 0.001, d = 2.722)]. In the CON group, there were no significant changes in physical quality test scores post-intervention. Relative changes from pre- to post-intervention indicated no significant differences in △-T-5m between INT and CON groups, while significant differences were found in other physical quality assessments [△-1RM bench press (3.376 vs 0.220 kg, p < 0.001), △-1RM squat (3.665 vs −0.533 kg, p < 0.001), △-T-10m (−0.005 vs 0.005 s, p = 0.004), △-T-30m (−0.153 vs −0.017 s, p < 0.001), △-CMJ (4.846 vs 0.615 cm, p < 0.001), △-RAT (−0.048 vs −0.002 s, p < 0.001), △-YBT-L (5.154 vs −0.615, p < 0.001), △-YBT-R (5.615 vs −0.308, p < 0.001)].

**Conclusion:**

A 12-week INT intervention significantly enhances the physical qualities of Sanda athletes. The overall improvements in the INT group across 1RM, 30MST, CMJ, RAT, and YBT tests demonstrate that the INT program may serve as an effective conditioning strategy for improving selected physical fitness outcomes in elite Sanda athletes.

## 1 Introduction

Martial arts sanda, also known as martial arts sanshou, is a branch of martial arts that employs various offensive and defensive techniques, including striking, kicking, and grappling, to overcome opponents in a highly competitive, unarmed combat setting [1]. This sport demands exceptional physical attributes, such as agility, flexibility, stability, and neuromuscular control. However, due to the specificity of its technical movements, training content, and competition requirements, the incidence of sports injuries is notably high [2,3]. To secure victory in competitions, athletes must adeptly select relevant stimuli to anticipate attacks and determine optimal counteractions against opponents, highlighting the critical importance of enhancing athletes’ perceptual and cognitive abilities [4]. As the Sanda discipline continues to evolve, competition has accelerated, necessitating athletes to perform spontaneous, high-frequency technical maneuvers along with increased foot movement and tactical transitions. Consequently, agility has emerged as a vital factor influencing both the development of Sanda and the determinants of competitive success [5]. Excellent physical fitness not only ensures that athletes can execute complex techniques and enhance the quality of their performance but also that flexibility and stability allow for unrestricted movement and prevent excessive compensation during actions. This contributes to the efficient execution of technical movements, effectively reduces the risk of injuries, and plays a significant role in extending an athlete's career [2,6]. In addition to agility, balance, and neuromuscular control, strength, and explosive power are equally crucial for athletic performance [7]. Elite athletes often encounter performance bottlenecks [8], and after prolonged periods of intense training, they may experience sports injuries and psychological burnout. This underscores the necessity for efficient training methods or schedules to address performance obstacles and enhance overall physical fitness and athletic performance [9].

Integrative Neuromuscular Training (INT) is recognized as a comprehensive training approach aimed at enhancing athletic performance and preventing sports injuries. It encompasses a combination of general foundational functional movement training and specific strength, plyometric, speed, agility, and balance training. INT programs have been shown to significantly improve muscle recruitment patterns, postural stability, and dynamic lower limb balance [10]. This training intervention also seeks to develop athletes’ perceptual and cognitive abilities during physical activities, offering a more refined and precise methodology compared to traditional, broad-based physical conditioning [11–13]. While injury prevention remains a key objective, coaches and athletes primarily favor the adoption of INT programs to enhance athletic performance [14]. Currently, INT training methods have been widely implemented across various sports, including football [15], basketball [16], volleyball [17], floorball [18], and table tennis [19], to improve athletes’ physical conditioning. However, there is a notable lack of practical research on the application of Integrative Neuromuscular Training specifically for elite martial arts athletes engaged in Sanda.

This study aims to explore the effects of Integrative Neuromuscular Training (INT) on strength, speed, lower limb explosive power, reactive agility, coordination, and balance in elite male Chinese Sanda athletes through experimental intervention, thereby addressing this research gap. It seeks to investigate the relationship between INT and physical fitness, offering a solution for elite Sanda athletes to overcome performance bottlenecks in physical conditioning. Furthermore, this research intends to provide scientific evidence and practical guidance for enhancing athletes’ physical capabilities and performance while effectively preventing sports injuries.

## 2 Materials and methods

### 2.1 Participants

A total of 26 participants (2 international-level athletes, 3 national-level athletes, and 21 national first-level athletes) completed all tests in this study (see Table 1). The study employed a parallel design, with participants randomly assigned in a 1:1 ratio to either the experimental intervention group (INT, n = 13) or the control group (CON, n = 13). All participants were physically healthy, injury-free, non-smokers, and voluntarily participated in this study. Informed consent was obtained after they were briefed on the testing procedures and potential risks. Participants were recruited between 01/11/2024 and 29/11/2024. Participants were randomly assigned in a 1:1 ratio using a computerized random number generator with concealed allocation (sequentially numbered, sealed opaque envelopes) to ensure group concealment before baseline assessment. The research protocol was approved by the Sports Science Experiment Ethics Committee of Beijing Sport University (Approval No. 2023352H). The clinical trial titled ‘The Impact of Integrative Neuromuscular Training on the Physical Fitness of Elite Male Martial Arts Sanda Athletes’ was registered with the Chinese Clinical Trial Registry (ChiCTR) under registration number ChiCTR2400092688 on 21/11/2024. Participants were recruited from different competitive levels; however, random allocation was used and no significant between-group differences were observed in demographic characteristics or baseline performance variables prior to the intervention, which may have partly minimized the influence of participant heterogeneity on the between-group comparisons.

**Table 1:** Characteristics of Participants.

Indicators	INT(n = 13)	CON(n = 13)	P-value
Age (years)	20.6 ± 2	20.5 ± 2	0.746
Training experience (years)	8.7 ± 3	8.5 ± 3	0.461
Height (cm)	177.34 ± 15.73	178.26 ± 11.41	0.652
Body mass (kg)	72.19 ± 18.27	72.37 ± 20.43	0.639
BMI（kg/m^2^）	22.95 ± 2.15	22.77 ± 1.92	0.745

BMI = Body Mass Index.

INT: Integrative Neuromuscular Training Group; CON: Control Group.

### 2.2 Procedures

**Fig 1:**
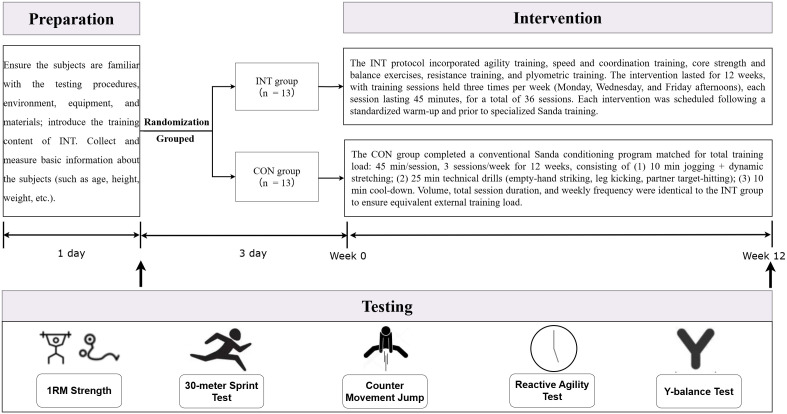
Experimental flowchart‌‌.

This study employed a randomized controlled trial design, consisting of four phases (see Fig 1). The first phase involved preparing the experimental setup, during which participants became familiar with all testing procedures, the testing environment, equipment, and the content of the Integrative Neuromuscular Training (INT). Basic participant information was collected and measured during this phase. The second phase was the first testing period, and the fourth phase was the second testing period. Both testing periods included assessments of one maximum repetition (1RM), a 30-meter sprint, counter-movement jump (CMJ), reactive agility, and Y-balance tests. The third phase constituted the intervention phase. A 72hour (3 day) recovery interval was implemented between baseline maximal exertion testing and the first intervention session to eliminate residual neuromuscular fatigue. During the 12-week intervention, both groups trained three times per week, with each session lasting 45 minutes and being performed after a standardized warm-up and before regular Sanda practice. The INT group completed a progressive integrative neuromuscular training program consisting of reaction and agility, speed and coordination, core stability and balance, strength, and plyometric modules. The CON group completed a conventional conditioning program routinely used in Sanda training, primarily including jogging, solo striking practice, and paired target-striking drills. Detailed week-by-week training content, loading, repetitions, and sets for both groups are now provided in Appendix S1.‌‌

### 2.3 Testing protocols and measurements

Before testing, all participants completed a standardized warm-up routine, as referenced in previous studies [20]. This routine included 3 minutes of low-intensity jogging followed by static stretching exercises targeting the quadriceps, hamstrings, gastrocnemius, gluteal muscles, and hip adductors, with each stretch held for 15 seconds. Subsequently, participants performed dynamic stretching using squats, leg swings, and other specific exercises typically employed by sprinters. To conclude the warm-up, participants executed two maximal-effort 30-meter sprints. Following the warm-up, all tests were conducted sequentially in an indoor facility, with participants permitted to sit quietly or walk slowly during the intervals between tests. All assessors were blinded to group allocation throughout pre‑ and post‑intervention testing to minimize detection bias*.*

#### 2.3.1 *1RM strength assessment.*

The One-Repetition Maximum (1RM) Strength Assessment is utilized to evaluate maximal strength. The testing protocol begins with participants performing squats using a predetermined weight that is below their estimated 1RM. The total weight is then progressively increased in increments of 1–10 kg based on the participant's feedback. During the squat, participants are required to maintain a fixed foot stance and toe angle, ensuring that the knees do not extend beyond the toes and that the thighs reach a parallel position to the ground [21]. A rest period of 2 minutes is allowed between consecutive trials. The final weight that a participant can lift for a single repetition is recorded as their 1RM. All participants successfully determined their 1RM after completing 3–5 trials. The procedure for the bench press test mirrors that of the squat, with participants instructed to lower the barbell to the chest before pressing it upward until their arms are fully extended [22].

#### 2.3.2 *30-meter sprint test.*

The 30-meter Sprint Test (30MST) is employed to assess speed performance. Following established protocols [20], the test is conducted on an indoor synthetic straight track, utilizing laser measurement equipment (LAVEG, Jenoptik, Sport) to acquire linear distance measurements at a sampling frequency of 100 Hz throughout all trials. The laser measurement procedures align with the recommendations set forth by Harrison et al. [23]. A 30-meter track is marked with a clearly defined starting line and finish line, with the laser mounted on a tripod at a height of 1.37 meters, positioned 2 meters behind the starting line. Participants are instructed to wear fitted white T-shirts to enhance the reflection of the laser signal. The laser is aimed at each participant's lower back. At the starting line, a research assistant signals the beginning of the test with a whistle, while another assistant records the time at the finish line using a stopwatch with a precision of 0.01 seconds. The testing procedure involves participants starting from a static squat position upon hearing the whistle signal, followed by three maximal effort 30-meter sprints, with a 2-minute rest interval between each trial. The shortest time recorded from the three trials is designated as the 30-meter sprint performance. Additionally, the times taken to reach 5 meters and 10 meters during the sprint are recorded, alongside the maximum speed achieved and the time taken to reach that maximum speed.

#### 2.3.3 *Counter movement jump.*

The Counter Movement Jump (CMJ) is utilized to reflect the explosiveness of the lower limbs. The testing procedure is as follows: participants stand with their hands placed on their hips on the Smartjump vertical jump mat (Smartjump, Fusion Sport, Australia) and perform a rapid squat to a knee flexion of 90 degrees while maintaining an upright posture. Subsequently, they execute a swift jump, during which participants are instructed to keep their torso as vertical as possible to minimize the influence of upper body movement on the test results. In this study, participants are required to complete a series of five consecutive CMJ tests, with the first two serving as warm-ups and the last three as official trials. Each CMJ test is separated by a 15-second recovery interval during which participants remain standing. During the tests, participants are instructed to jump with maximal effort, and the highest jump height from the final three trials is recorded for analysis [24].

#### 2.3.4 *Reactive agility test.*

The Reactive Agility Test (RAT) is employed to measure the quality of reaction agility, following established methodologies from previous research [25]. The procedure is as follows: participants stand 0.5 meters behind the Smartspeed timing gates (Smartspeed Pro, Fusion Sport, Australia) and, upon hearing the “Go” command, complete the Y-shaped reactive agility test as quickly as possible. This test consists of a 5-meter sprint followed by a 45° change of direction (either left or right) and then another 5-meter sprint. The direction of the change is randomly determined by a triggering device located 2.5 meters away (triggering timing gate). As participants pass through this device, the timing gates at the left and right endpoints are randomly illuminated, requiring participants to react swiftly and sprint through the illuminated timing gate. Each run from the starting point to the endpoint constitutes one test trial, with a total of six trials completed (three to the left and three to the right). A 30-second interval is provided between trials, during which participants jog or walk back to the starting point to prepare for the next test. The shortest sprint time from these trials is recorded for analysis [26,27].

#### 2.3.5 *Y-balance test.*

The Y-balance Test (YBT) is utilized to evaluate the balance capabilities of participants, following established methodologies from previous research [28]. The testing procedure is as follows: participants stand on the Y-Balance Test apparatus with their hands placed on their hips and one bare foot planted firmly on the platform. They then use the toes of the non-supporting leg to slowly push the testing boards in three directions to their maximal reach. The testing sequence involves first measuring with the right leg as the support leg, followed by the left leg, assessing the maximum distance achieved in the anterior (A), posteromedial (PM), and posterolateral (PL) directions. Each direction is measured three times, and the maximum distance reached in each direction is recorded for analysis. At the conclusion of the test, the comprehensive score for the participant's YBT is calculated based on the distances achieved in the three directions for both legs. The formula for calculating the composite value (%) is as follows:

Composite Value (%)=[(A + PM + PL) / (3×Lower Limb Length)]×100.

This score serves as an assessment of the participant's balance ability.

### 2.4 Intervention program design

In this intervention plan, the INT group utilized an INT protocol adapted from previous studies [12,15,29]. This protocol incorporated a combination of agility training, speed and coordination training, core strength and balance training, resistance strength training, and plyometric training. The intervention spans 12 weeks, with a training frequency of three sessions per week (Monday, Wednesday, and Friday afternoons), each lasting 45 minutes, totaling 36 intervention sessions. Each intervention session was conducted after a standardized warm-up, which was identical to the warm-up performed before testing [20], and before the specialized Sanda training.

The INT program is structured to adjust and increase the training content, intensity, and volume every three weeks (Appendix S1). During weeks 1, 4, 7, and 10, particular emphasis is placed on the accuracy of individual movements, the active range of motion, and the quality of movement completion. Adjustments and increases adhere to a progressive principle, transitioning from basic bodyweight exercises to those incorporating additional equipment such as medicine balls, from static balance exercises to dynamic balance training, and from single-module balance training to multi-module training that includes reactive and coordination modules. The training load progressively increases over time. This incremental approach to training difficulty, volume, and accumulation emphasizes neural adaptation as well as the development of muscular endurance and strength [30,31].

The training model follows a circuit-style format; for instance, in the initial three weeks, participants complete each module (e.g., strength training) before proceeding to the next module (e.g., plyometric training). Specific training modalities (e.g., 70% 1RM Split Squat) are practiced after completing all prescribed repetitions and sets before transitioning to the next modality (e.g., 70% 1RM Bench Press). Rest intervals of 20–120 seconds are provided between sets and exercises. All participants underwent 1RM testing for squat and bench press before the training sessions [30], and the loading parameters for strength exercises were based on recommendations from prior studies [32,33].

The CON group completed a conventional Sanda conditioning program matched for total training load: 45 min/session, 3 sessions/week for 12 weeks, consisting of (1) 10 min jogging + dynamic stretching; (2) 25 min technical drills (empty‑hand striking, leg kicking, partner target‑hitting); (3) 10 min cool‑down. Volume, total session duration, and weekly frequency were identical to the INT group to ensure equivalent external training load. We also added Appendix S1 with week‑by‑week progression for both INT and CON groups.

### 2.5 Statistical analyses

Statistical analyses were conducted using SPSS (version 25.0; SPSS Inc., Armonk, NY). The Shapiro-Wilk test was employed to assess the normality of data distribution for each group. For data that violated the normality assumption as indicated by the Shapiro-Wilk test, the Mann-Whitney U test was utilized. Descriptive statistics were presented as mean ± standard deviation (M ± SD). Independent samples t-tests were performed to determine whether significant differences existed in demographic characteristics, baseline features, and relative changes (∆) in test metrics from PRE to POST between the intervention group (INT) and control group (CON). For between group independent t tests/Mann–Whitney U tests, a Bonferroni correction was applied to control Type I error due to multiple dependent variables. For data that did not meet the normality assumption, the Wilcoxon signed-rank test was executed to compare PRE and POST values. Additionally, Cohen’s d effect sizes were reported for paired sample t-tests, with interpretation criteria for d as follows: < 0.2 (small), 0.2–0.6 (medium), 0.6–1.2 (large), 1.2–2.0 (very large), and >2.0 (extremely large) [34]. Significance levels were set at P < 0.05 for statistical significance, P < 0.01 for high significance, and P < 0.001 for very high significance. A post hoc power analysis was conducted using G*Power (version 3.1) based on the primary outcome (countermovement jump, CMJ) with α = 0.05. The achieved power was 0.82, indicating the sample size (n = 13 per group) provided sufficient statistical power to detect the observed between group differences.

## 3 Results

Although participants were recruited from international, national, and national first‑level competitive categories, randomized 1:1 allocation was applied, and no significant between‑group differences were detected in demographic or baseline performance variables (all p > 0.05), which minimized confounding from heterogeneity. Nevertheless, residual heterogeneity cannot be fully excluded due to the relatively small sample (as shown in Table 1).

No significant differences were observed between the intervention group and the control group in any of the test metrics before the intervention (all p > 0.05), indicating no baseline differences between the groups (as shown in Table 2).

**Table 2:** Baseline Comparison of INT and CON Groups Before Intervention.

Indicators	INT(n = 13)	CON(n = 13)	P-value
1RM bench press (kg)	74.34	74.07	0.862
1RM squat (kg)	96.14	96.80	0.648
T-5m (s)	1.495	1.494	0.895
T-10m (s)	2.168	2.166	0.887
T-30m (s)	4.043	4.025	0.731
CMJ (cm)	44.85	44.46	0.830
RAT (s)	2.182	2.180	0.911
YBT-L	94.15	95.23	0.624
YBT-R	94.31	95.54	0.646

INT: Integrative Neuromuscular Training Group; CON: Control Group.

Post-intervention, the INT group showed significant improvements in these physical fitness measures: [1RM bench press (p < 0.001, d = 2.599), 1RM squat (p < 0.001, d = 2.610), T-5m (p = 0.011, d = −1.000), T-10m (p = 0.011, d = −0.833), T-30m (p < 0.001, d = −6.120), CMJ (p < 0.001, d = 4.236), RAT (p < 0.001, d = −3.312), YBT-L (p < 0.001, d = 3.075), YBT-R (p < 0.001, d = 2.722)] (see Table 3). In the CON group, there were no significant changes in physical quality test scores post-intervention. Relative changes from pre- to post-intervention indicated no significant differences in △-T-5m between INT and CON groups, while significant differences were found in other physical quality assessments [△-1RM bench press (3.376 vs 0.220 kg, p < 0.001), △-1RM squat (3.665 vs −0.533 kg, p < 0.001), △-T-10m (−0.005 vs 0.005 s, p = 0.004), △-T-30m (−0.153 vs −0.017 s, p < 0.001), △-CMJ (4.846 vs 0.615 cm, p < 0.001), △-RAT (−0.048 vs −0.002 s, p < 0.001), △-YBT-L (5.154 vs −0.615, p < 0.001), △-YBT-R (5.615 vs −0.308, p < 0.001)] (see Table 4).

**Table 3:** Comparison of Physical Fitness Indicators Pre- and Post-Intervention in INT and CON Groups.

Indicators	Group	P	t	Means of differences	Cohen's d	95% CI
1RM bench press (kg)	INT	<0.001	9.370	3.376	2.599	2.591 ～ 4.161
	CON	0.344	0.985	0.220	0.273	−0.267 ～ 0.707
1RM squat (kg)	INT	<0.001	9.414	3.665	2.610	2.816 ～ 4.513
	CON	0.169	1.465	−0.533	−0.406	−1.326 ～ 0.260
T-5m (s)	INT	0.011	3.004	−0.003	−1.000	−0.005 ～ −0.001
	CON	0.656	0.456	0.001	0.125	−0.004 ～ 0.006
T-10m (s)	INT	0.011	3.010	−0.005	−0.833	−0.009 ～ −0.001
	CON	0.089	1.849	0.005	0.455	−0.001 ～ 0.012
T-30m (s)	INT	<0.001	22.41	−0.153	−6.120	−0.168 ～ −0.138
	CON	0.068	2.008	−0.017	−0.567	−0.035 ～ 0.001
CMJ (cm)	INT	<0.001	15.28	4.846	4.236	4.155 ～ 5.537
	CON	0.165	1.477	0.615	0.410	−0.292 ～ 1.523
RAT (s)	INT	<0.001	11.94	−0.048	−3.312	−0.057 ～ −0.040
	CON	0.513	0.674	−0.002	−0.187	−0.010 ～ 0.005
YBT-L	INT	<0.001	11.090	5.154	3.075	4.141 ～ 6.166
	CON	0.104	1.760	−0.615	−0.488	−1.377 ～ 0.147
YBT-R	INT	<0.001	9.814	5.615	2.722	4.369 ～ 6.862
	CON	0.367	0.9385	−0.308	−0.260	−1.022 ～ 0.407

**Table 4:** Relative Changes of Test Indicators Pre- and Post-Intervention in INT and CON Groups.

Indicators	p	t	The mean of △-INT	The mean of △-CON	95% CI
△-1RM bench press (kg)	<0.001	7.446	3.376	0.220	−4.031 ～ −2.281
△-1RM squat (kg)	<0.001	7.876	3.665	−0.533	−5.298 ～ −3.098
△-T-5m (s)	0.141	1.521	−0.003	0.001	−0.001 ～ 0.009
△-T-10m (s)	0.004	3.151	−0.005	0.005	0.004 ～ 0.018
△-T-30m (s)	<0.001	12.55	−0.153	−0.017	0.114 ～ 0.159
△-CMJ (cm)	<0.001	8.080	4.846	0.615	−5.311 ～ −3.150
△-RAT (s)	<0.001	8.690	−0.048	−0.002	0.035 ～ 0.057
△-YBT-L	<0.001	9.920	5.154	−0.615	−6.970 ～ −4.569
△-YBT-R	<0.001	8.981	5.615	−0.308	−7.284 ～ −4.562

## 4 Discussion

The results of this study suggest that a 12-week Integrative Neuromuscular Training (INT) program has a beneficial impact on the overall performance of martial arts Sanda athletes. Specifically, participants in the INT group showed improvements in strength, speed, jumping ability, reaction time, and balance. Furthermore, INT appears to be a safe training intervention, as no injuries were reported during implementation, despite the gradual increases in training intensity, volume, and duration.

In this study, the INT training program demonstrated a significant positive effect on the strength (1RM), movement speed, and vertical jump height of Sanda athletes. These findings align with previous INT research, which has shown significant improvements in 1RM following various INT interventions, whether conducted twice, three times, or four times a week over an 8-week period [15,19,35]. Notably, after 8 weeks of INT training twice a week, the time for a 10-meter sprint was significantly reduced [35,36]. Additionally, a 19.1 ± 4.2 cm increase in Countermovement Jump (CMJ) was observed (19.1 ± 4.2 vs. 20.3 ± 4.04 cm, d = 0.3) after 8 weeks of INT training [15]. Furthermore, after 6 weeks of INT training, vertical jump height increased from 39.9 ± 0.9 cm to 43.2 ± 1.1 cm [37]. Improvements in sprint speed and reaction time during rapid changes of direction were also noted after 6 weeks of INT training conducted twice a week [38]. Finally, following 12 weeks of INT training, significant improvements were observed in single-leg balance (OR=2.8; 95% CI 1.1 to 4.6) and the Star Excursion Balance Test (SEBT) (OR=4.7; 95% CI 2.2 to 7.1) [39].

Previous research has reported a strong correlation between leg extensor strength and sprint performance, indicating that increases in lower limb muscle strength can enhance short-duration sprint capabilities [40]. Integrative Neuromuscular Training (INT) typically enhances vertical jump performance by improving leg muscle strength [41,42]. Furthermore, vertical jump height has been shown to be significantly associated with sprinting ability [43,44]. Enhancements in sprint performance and muscle strength can also lead to improvements in agility, particularly in change of direction (CoD) performance [45]. Additionally, a significant correlation exists between eccentric strength and dynamic balance, with moderate correlations observed between the ability to generate power and dynamic balance [46]. These findings further corroborate the positive improvements in strength, speed, vertical jump height, and agility observed in this study.

The observed positive improvements in strength, speed, vertical jump height, agility, and balance can be attributed to various factors inherent in the training content selected for this study. The INT program integrates diverse modalities of strength, speed, power, agility, core strengthening, balance, resistance training, and plyometric training. This comprehensive approach combines basic and specific movements, featuring both repetitive training of individual technical skills and multifaceted training of various athletic qualities. The enhancement in physical fitness among participants likely results from the strategic combination of different technical movements and the synergistic effects of the diverse training interventions employed.

For instance, numerous studies and meta-analyses have demonstrated the positive impact of plyometric training—incorporated within our INT programming strength, speed (e.g., Countermovement Jump), and reactive performance. Research comparing 12 weeks of enhanced training, resistance training, and a combination of both found that the combined training approach significantly outperformed single-method training regarding improvements in athletes’ leg strength [47]. Our INT program effectively integrates extensive content from both strength training and plyometric training, encompassing both full-body exercises (e.g., squats) and specific lower limb muscle training (e.g., prone leg curls with machines).

Moreover, the plyometric jump training included in the INT program comprises various jump exercise variations, all aimed at enhancing athletes’ lower limb strength and power [48]. Miller et al. observed significant improvements in performance on the Illinois agility test in athletes following 6 weeks of plyometric training [49]. Participants exhibited small to moderate improvements (Effect Size [ES] = 0.35–0.80, all p < 0.05) in most outcomes, including maximal dynamic strength, linear sprint speed, horizontal jump distance, and reactive strength index, after engaging in plyometric jump training [50]. A systematic review with meta-analysis focused on combat athletes indicated that plyometric-jump training programs, spanning 4–12 weeks and comprising 2–3 sessions per week, yielded small to moderate improvements (ES = 0.47 to 1.04) in maximal strength (e.g., 1RM squat), vertical jump height, and change-of-direction speed when compared to control groups [51]. MYER et al. concluded that enhanced training and dynamic balance exercises significantly improve biomechanical and neuromuscular performance, as well as balance ability [52].

Additionally, a study involving football players demonstrated that forward lunge training—conducted twice a week for 6 weeks—resulted in significant improvements in both hamstring strength and sprint performance for participants in both the walking forward lunge and jumping forward lunge groups [53]. Another study showed that in-line lunge movements correlate positively with agility performance [54], which aligns with the inclusion of similar lunge training elements in our INT program.

According to Sheppard and Young [55], agility is defined as a rapid whole-body movement that involves a change in velocity or direction in response to a stimulus. As such, agility encompasses both a change of direction (CoD) component [56] and a perceptual decision-making component. The agility training integrated into this INT program, including exercises such as Rapid Dodge and Counterattack Training, likely enhanced the cognitively determined abilities of the Sanda athletes. This training may have facilitated greater motor unit recruitment and synchronization, as well as an increased rate of coding [57], ultimately fostering an adequate neuromuscular response.

Numerous factors influence the maintenance of balance, including the eccentric strength of the knee extensors, lower limb explosive strength, core stability, and the range of motion in lower limb joints [46,58]. To address these factors, our INT program incorporates training components designed to enhance eccentric explosive strength of the knee extensors, such as plyometric jumps and forward lunges. Additionally, it includes core strength training exercises, such as Russian twists with a medicine ball and side bridges, which are aimed at increasing the strength of the abdominal and spinal muscles, thereby improving core stability. Furthermore, lower limb strength training is crucial for enhancing hamstring strength, which is vital for the stability of the knee joint [59].

Furthermore, the gains in explosive strength, speed, and balance may be attributed to neuromuscular adaptations, such as more effective motor unit recruitment, rate coding (the frequency of action potentials), synchronization, and intermuscular coordination [60,61]. Research has demonstrated that INT training enhances the synchronization and coordination of muscle activity patterns in athletes, thereby improving dynamic joint stability and fine motor control, which in turn enhances movement efficiency [62,63]. Speed improvements may be linked to increased neuromuscular activation, specifically the upregulation of motor unit discharge frequency, as well as improvements in ground contact time and the stiffness of muscle-tendon units [36,64].

More specifically, the increase in the number of active motor units or their firing frequency, along with changes in the recruitment patterns of motor units (primarily fast-twitch fibers), may contribute to enhanced speed [65]. These factors could accelerate the development of explosive strength [66], maximize force production [67], and improve the efficiency of the stretch-shortening cycle [68], potentially enhancing sprinting performance through changes in stride length and frequency [69].

Some studies suggest that balance training improves postural stability by diminishing the potential for Ia afferent nerve activity to exert inhibitory effects through intermediate inhibitory neurons; this spinal cord response inhibition may help improve post-training balance performance [70]. Additionally, our INT program includes multidirectional movements (e.g., simulating agility T-tests and lateral rope ladder training), which may enhance adaptability in both the peripheral and central nervous systems, thereby increasing proprioceptive awareness in the lower limbs [71]. Consequently, the combined effects of training for core strength, lower limb explosive power, and proprioception manifest as improved Y-balance scores.

Additionally, the training content within this INT program shares similarities with the testing components that follow. The researchers believe this aligns with the principle of training specificity, which posits that when the characteristics of training (such as exercise type, contraction pattern, and movement speed) are consistent with the activities being tested, the adaptations related to training are likely to be more pronounced [72].

The muscle strength and explosiveness of both the upper and lower limbs are crucial for Sanda athletes, as most technical movements in combat sports originate from the lower body. Compared to the upper limbs, the significance of lower limb strength and power is particularly pronounced [73]. Research has indicated that the correlations between strength/power variables and punching impact indices range from 0.67 to 0.85, highlighting strong associations between punching impact and strength/power variables, especially lower limb muscle power [7]. Additionally, studies have found that 1RM squat strength can serve as an important indicator for distinguishing the neuromuscular qualities of higher and lower-level mixed martial arts competitors [74]. This view is supported by Lachlan P. James, who asserts that neuromuscular strength and anaerobic capacity can differentiate high-level from low-level combat athletes, noting that those with better lower limb strength and explosiveness are likely to execute technical movements more effectively, thereby enhancing athletic performance [75]. In Sanda competitions, the fighting area measures 8 meters by 8 meters, making movement speed equally vital for athletes. In particular, the ability to move quickly within a 5-meter radius is essential, as athletes need to swiftly position themselves in front of their opponents to deliver effective strikes, evade attacks, and prepare for subsequent offensive actions.

The INT training program in this study is beneficial for improving the neuromuscular adaptability and excitability of motor neurons in Sanda athletes. The Hoffman (H) reflex can be utilized to assess the excitability of spinal α-motoneurons while also reflecting transmission efficiency, particularly presynaptic inhibition in Ia afferent synapses [76]. The H-reflex is best interpreted as an estimate of “net spinal excitability,” which encompasses the summation of both excitatory and inhibitory descending and afferent synaptic inputs, presynaptic inhibition, and the intrinsic properties of motor neurons [77]. Pre-reaction time refers to the duration taken to identify external stimulus signals and respond appropriately before entering the execution phase [78]. Research has demonstrated that after 14 weeks of resistance training, an increase in maximum muscle strength during contractions is accompanied by an enhancement in the H-reflex response ^[^76^]^. This may indicate that INT training improves the excitability of α-motoneurons in Sanda athletes, enabling them to process stimulus signals more rapidly in the brain. Consequently, commands are transmitted via efferent neurons to effectors, stimulating the corresponding muscles to contract and generate movement [79].

This improvement may also be a result of neuromuscular adaptation to external signals, which is crucial for athletes during competition. In high-intensity matches, athletes must quickly assess the status of their opponents to seize opportunities for precise attacks. During defensive maneuvers, observing the opponent's attacking patterns allows athletes to anticipate and evade or block incoming strikes, enabling them to respond with counterattacks effectively.

### 4.1 Limitations of the study

A limitation of the present study is that the participants were drawn from different competitive levels, which may have introduced some heterogeneity in baseline training background and responsiveness to the intervention. Although no significant between-group differences were observed at baseline, the relatively small sample size means that the potential influence of this heterogeneity cannot be fully excluded. In addition, the current study did not evaluate whether the INT intervention could be utilized for injury prevention or whether its benefits could translate into improved long-term health outcomes for athletes. Future research should specifically address these objectives through longitudinal studies. Furthermore, genetic variability may partly contribute to inter-individual differences in training responsiveness and performance adaptation. Future studies may therefore incorporate candidate genetic markers [80] or polygenic approaches to better understand whether adaptations to INT differ across athletes with different genetic profiles [81].

## 5 Conclusion

The 12-week INT intervention significantly improved the physical performance of the athletes. The enhancements observed in the INT group across measures such as 1RM, sprinting speed, Counter Movement Jump (CMJ), reaction agility, and Y balance tests demonstrate the feasibility of INT as a comprehensive training approach for elite Sanda athletes. Furthermore, INT may be incorporated as a regular conditioning strategy before formal training sessions to enhance selected aspects of physical performance in elite Sanda athletes.

### 5.1 Practical applications

Compared to traditional training methods, the INT training program places a stronger emphasis on developing agility, speed, neuromuscular coordination, proprioceptive control, stability, and lower limb explosiveness. Research has shown that the comprehensive improvements in physical qualities following the training support this emphasis [15,35]. This study structured the loading patterns according to the development principles of different physical qualities, employing lower loads for reaction, agility, and speed training while utilizing higher loads for resistance and plyometric training. The focus was on enhancing the athletes’ reaction agility, speed coordination, balance stability, and explosiveness. The experimental results confirm that the 12-week INT training program effectively achieved the expected goals of comprehensive physical improvement in elite Sanda athletes. This outcome indicates that, despite the participants’ already high physical quality levels, our designed INT training plan successfully enhanced their physical capabilities. It suggests to coaches and elite Sanda athletes that INT may have a positive impact on selected physical attributes, even in high-level competitors.
